# Mechanical Stretch Promotes the Osteogenic Differentiation of Bone Mesenchymal Stem Cells Induced by Erythropoietin

**DOI:** 10.1155/2019/1839627

**Published:** 2019-07-07

**Authors:** Yong-Bin He, Sheng-Yao Liu, Song-Yun Deng, Li-Peng Kuang, Shao-Yong Xu, Zhe Li, Lei Xu, Wei Liu, Guo-Xin Ni

**Affiliations:** ^1^School of Sport Medicine and Rehabilitation, Beijing Sport University, China; ^2^Department of Orthopedics, The Fifth Affiliated Hospital of Zunyi Medical University, China; ^3^Department of Orthopedics, The Second Affiliated Hospital of Guangzhou Medical University, China; ^4^Department of Orthopeadics and Traumatology, Nanfang Hospital, Southern Medical University, China; ^5^Department of Orthopaedics and Traumatology, Zhengzhou Orthopaedics Hospital, Zhengzhou, China; ^6^Department of Orthopedics, The People's Hospital of Gaoming District of Foshan City, China

## Abstract

**Introduction:**

The effects of erythropoietin (EPO) on the behaviors of bone marrow mesenchymal stem cells (BMSCs) subjected to mechanical stretch remain unclear. This study was therefore aimed at establishing the dose-response effect of EPO stimulation on rat BMSCs and investigating the effects of mechanical stretch combined with EPO on the proliferation and osteogenic differentiation of BMSCs.

**Material and Methods:**

The proliferation and osteogenic differentiation of rat BMSCs were examined and compared using EPO with different concentrations. Thereafter, BMSCs were subjected to 10% elongation using a Flexcell strain unit, combined with 20 IU/ml EPO. The proliferation of BMSCs was detected by Cell Counting Kit-8, colony formation assay, and cell cycle assay; meanwhile, the mRNA expression levels of Ets-1, C-myc, Ccnd1, and C-fos were detected by reverse transcription and real-time quantitative PCR (qPCR). The osteogenic differentiation of BMSCs was detected by alkaline phosphatase (ALP) staining, and the mRNA expression levels of ALP, OCN, COL, and Runx2 were detected by qPCR. The role of the extracellular signal-regulated kinases 1/2 (ERK1/2) in the osteogenesis of BMSCs stimulated by mechanical stretch combined with 20 IU/ml EPO was examined by Western blot.

**Results:**

Our results showed that effects of EPO on BMSCs included a dose-response relationship, with the 20 IU/ml EPO yielding the largest. Mechanical stretch combined with 20 IU/ml EPO promoted proliferation and osteogenic differentiation of BMSCs. The increase in ALP, mineral deposition, and osteoblastic genes induced by the mechanical stretch–EPO combination was inhibited by U0126, an ERK1/2 inhibitor.

**Conclusion:**

EPO was able to promote the proliferation and osteogenic differentiation of BMSCs, and these effects were enhanced when combined with mechanical stretch. The underlying mechanism may be related to the activation of the ERK1/2 signaling pathway.

## 1. Introduction

Large bone defects resulting from trauma, congenital defects, neoplasm, failed arthroplasty, and infection are quite common [1, 2], and the incidences of nonunion and delayed union are very high [3]. It remains a great challenge for orthopedic surgeons to achieve osseous reconstruction for nonunion and bone defects. Distraction osteogenesis (DO) is regarded as one of the most effective therapeutic strategies for posttraumatic complex nonunion [4–7]; but the overall therapeutic process lasts for a relatively long period, and a variety of complications may arise, such as pin loss, infection around the transmucosal pin, bone fracture, and restriction in joint motion [8]. Various approaches have been tested to promote bone formation in order to shorten the DO period. Among them, bone morphogenic proteins (BMPs) are thought to be the most potent osteoinductive factors and play a key role in the process of bone formation during DO. However, the high cost and the short half-life *in vivo* restrict usage of their applications [9–12]. It is therefore necessary to seek alternatives with good therapeutic outcomes for use in the DO technique.

Erythropoietin is a glycoprotein hormone that stimulates red blood cell (RBC) production in bone marrow via binding to the cell-surface receptor on hematopoietic progenitor cells, and it has been widely used for treating anemia [13]. In addition to its classical role in the regulation of RBC proliferation, EPO has been shown to exert protective and regenerative capabilities in a variety of nonhematopoietic tissues [14]. Notably, it promotes the osteogenic differentiation of bone mesenchymal stem cells (BMSCs) [15–17]. Furthermore, mechanical stretch may induce the differentiation of BMSCs into mature osteoblasts and enhance the deposition of the bone matrix [18–20]. Nevertheless, the effects of EPO remain unknown on the behaviors of BMSCs subjected to mechanical stretch, which is an *in vitro* condition simulated to an *in vivo* DO procedure.

This study was therefore aimed at addressing three key issues: First, is there a dose-dependent effect of EPO on the proliferation and osteogenic differentiation of BMSCs? And which is the optimal concentration if so? Second, what is the effect of EPO at its optimal concentration on the proliferation and osteogenic differentiation of BMSCs subjected to mechanical stretch? Third, is the ERK1/2 signaling pathway involved in the promotion of the osteogenic differentiation of EPO combined with mechanical stretch on BMSCs?

## 2. Material and Methods

### 2.1. BMSC Isolation, Identification, and Culture

This study was approved by the Animal Ethics Committee of Nanfang Hospital, Southern Medical University. The BMSCs were isolated from 4-week-old male Sprague-Dawley rats using a method described previously [21]. The morphology of the cells was observed by optic and inverted phase contrast microscope. After identification by flow cytometry, the third generation (P3) cells were selected for further study. L-DMEM containing 10% fetal bovine serum (FBS) was used as the culture medium.

Flow cytometry analysis and cell cycle assay were conducted using a method described in our previous study [21]. Briefly, the isolated cells were collected and incubated with antibodies of CD11b, CD45, CD79, and CD90 according to the manufacturer's instructions. After being incubated for 30 minutes at 4°C, the cells were analyzed by flow cytometry (BD Biosciences, USA). The isolated cells were then collected and fixed in 70% precooling ethanol overnight at 4°C. After being stained with propidium iodide (PI)/RNase buffer at room temperature in the dark for 30 minutes, the isolated cells were analyzed for cell cycle by flow cytometry.

### 2.2. Experimental Design

After incubation for 24 hours, the cells were treated with EPO with different concentrations of rhEPO (5 IU/ml, 10 IU/ml, 20 IU/ml, and 40 IU/ml). Cells untreated with rhEPO were defined as the control group. After 7 days of being cultured, the cells were examined for their proliferation and osteogenic differentiation. Thereafter, the optimal concentration of rhEPO was chosen for further investigations.

After being incubated for 24 hours, the cells were treated with EPO and/or mechanical stretch and divided into the following 4 groups: the control (CON) group, EPO group, stretch (STR) group, and EPO+STR group. In terms of mechanical stretch, a cyclic mechanical stretch with a 1 Hz sinusoidal curve set at 10% elongation was applied for 4 hours twice a day using the FX-5000TM Flexcell Tension Plus™ unit (Flexcell International Corporation, NC, USA). After treatment for 7 days, CCK-8 was applied to the cells to detect proliferation. The colony formations and the cell cycles were examined by colony formation assay (CFA) and flow cytometry, respectively. The osteogenic differentiation of the cells was detected by ALP staining and ALP activity. Reverse transcription and real-time qPCR were used to examine the gene expressions of Ets-1, C-myc, Ccnd1, C-fos, ALP, OCN, COL, and Runx2.

The cells were divided into 4 groups based on treatment methods: CON, EPO, STR, and EPO+STR. After 7 days of treatment, the expression levels of t-ERK1/2 and p-ERK1/2 were detected by Western blot. In addition, the P3 cells were further divided into 4 groups based on treatment methods: CON, STR+EPO, CON+blocking, STR+EPO+blocking. An osteogenic-induced medium was used to induce cell osteogenesis, whereas U0126 (10 *μ*M) was used as an ERK1/2 blocker. After 7 days of treatment, the osteogenic differentiation of the cells was detected by ALP activity.

### 2.3. Cell Proliferation

Cell Counting Kit-8 (CCK-8) assay was used to assess cell proliferation. In brief, all cells were harvested and incubated with CCK-8 solution according to the manufacturer's instructions. After being incubated for 2 hours at 37°C, the optical density (OD) value of the cells was measured at 450 nm with a microplate reader (SpectraMax M5, Molecular Devices, USA).

### 2.4. CFA Assessment

Approximately 1 × 10^3^ cells from each group were seeded in 3 wells of a 6-well culture plate and incubated under the same conditions noted above for 14 days. After incubation, the medium was removed by aspiration and the colonies were washed with PBS. The colonies were fixed with 4.0% (*w*/*v*) paraformaldehyde for 20 minutes, then stained with 0.1% crystal violet for 1 hour at room temperature. After washing excess crystal violet with ddH_2_O, the plates were left to dry in normal air at room temperature overnight.

### 2.5. ALP Activity and Staining Measurement

ALP activity and staining was performed according to a previous protocol [22]. For the ALP activity assay, cells from each group were rinsed twice with PBS, then lysed with 10 mM Tris–HCl containing 2 mM MgCl_2_ and 0.05% Triton X-100 (pH 8.2) at 4°C. Sonicated cell lysates were subsequently centrifuged at 12000g for 10 min at 4°C, then the supernatants were used for the assays. Lysates were incubated in an ALP detection buffer for 30 min at 37°C. The reaction was stopped by adding 0.1 M NaOH and then monitored at 405 nm. Total protein was measured spectrophotometrically using a Micro BCA Protein Assay Kit (Pierce) and read at 562 nm. The enzymatic activity of ALP was normalized to the total protein content of the sample (405/562 nm). For the ALP staining assay, cells from each group were fixed with 4% paraformaldehyde for 15 min, stained with ALP detection solution for 30 min at 37°C, and washed with PBS to remove excess staining.

### 2.6. qPCR

Total RNA was extracted from cells both in the experiment group and in the control group using RNAiso Plus (Takara Bio Inc., Dalian, China). RNA purity and concentration were quantified spectrophotometrically using a NanoDrop ND-1000 (NanoDrop Technologies, USA). The cDNA (10 *μ*l final volume) was synthesized from the total RNA using the Bestar qPCR RT Kit (DBI Bioscience, Germany) according to the manufacturer protocol. The qPCR reactions (20 *μ*l final volume) were conducted using the Bestar SybrGreen qPCR Mastermix (DBI Bioscience, Germany). The primer sequences for these experiments are listed in Table 1.

The cDNA was amplified in triplicate at 95°C for 2 minutes, followed by 40 denaturation cycles at 95°C for 10 seconds, annealing and extension at 60°C for 30 seconds, and 40 more denaturation cycles at 72°C for 30 seconds. A dissociation curve was constructed to confirm that there was no nonspecific amplification. Glyceraldehyde-3-phosphate dehydrogenase (GAPDH) was quantified as an endogenous reference, and each sample was normalized to its GAPDH content. Dissociation curve analysis was used to determine the specificity of qPCR. The data from qPCR experiments was analyzed using the comparative CT method as described in the manual for the LightCycler® 480 System (Roche Life Science, Canada) to determine relative quantitative gene expression.

### 2.7. Western Blot Analysis

After 7 days of treatment, the cells from 4 groups were washed with ice-cold PBS and digested immediately. The proteins were extracted using a cell lysis buffer. After electrophoretic separation by 8% SDS-polyacrylamide gel electrophoresis, the proteins were electrotransferred onto polyvinylidene fluoride (PVDF) membranes (Millipore, Billerica, MA, USA). The membranes were then blocked with Tris-buffered saline containing 0.1% Tween-20 (TBST) and 5% skim milk for 1 hour at room temperature. t-ERK1/2 rabbit mAb, phospho-ERK1/2 (p-ERK1/2) rabbit mAb (Cell Signaling Technology, Danvers, MA, USA), and GAPDH rabbit mAb (4A Biotech, Beijing, China) were used according to the manufacturers' protocols, and the membranes were incubated overnight with these antibodies at 4°C with slight shaking. Thereafter, the membranes were washed 3 times in TBST and further incubated with an HRP-conjugated antibody (goat anti-rabbit IgG, Sigma-Aldrich, Saint Louis, MO, USA) for 1 hour at room temperature. Finally, the membranes were washed 3 times in TBST, and the signals were developed using an enhanced chemiluminescence kit (KeyGen Biotech, Nanjing, China). A semiquantitative evaluation of the bands was performed by densitometry (VersaDoc, Bio-Rad, Hercules, CA, USA). The levels of t-ERK1/2 and p-ERK1/2 proteins were determined through their normalization to the protein level of GAPDH.

For inhibition of ERK1/2 activation, BMSCs were incubated with U0126 (10 *μ*M, Sigma-Aldrich, Saint Louis, MO, USA) for 30 minutes at 37°C before they were subjected to cyclic mechanical stretch combined with 20 IU/ml EPO.

### 2.8. Statistical Analysis

Data was statistically analyzed using SPSS 19.0 software. All experimental data were expressed as the means ± standard deviation. Differences between 2 groups were determined statistically by Student's *t*-test, and the variance was analyzed. One-way ANOVA with post hoc LSD was performed, and *p* values < 0.05 were considered statistically significant.

## 3. Results

### 3.1. Cell Characterization

**Figure 1:**
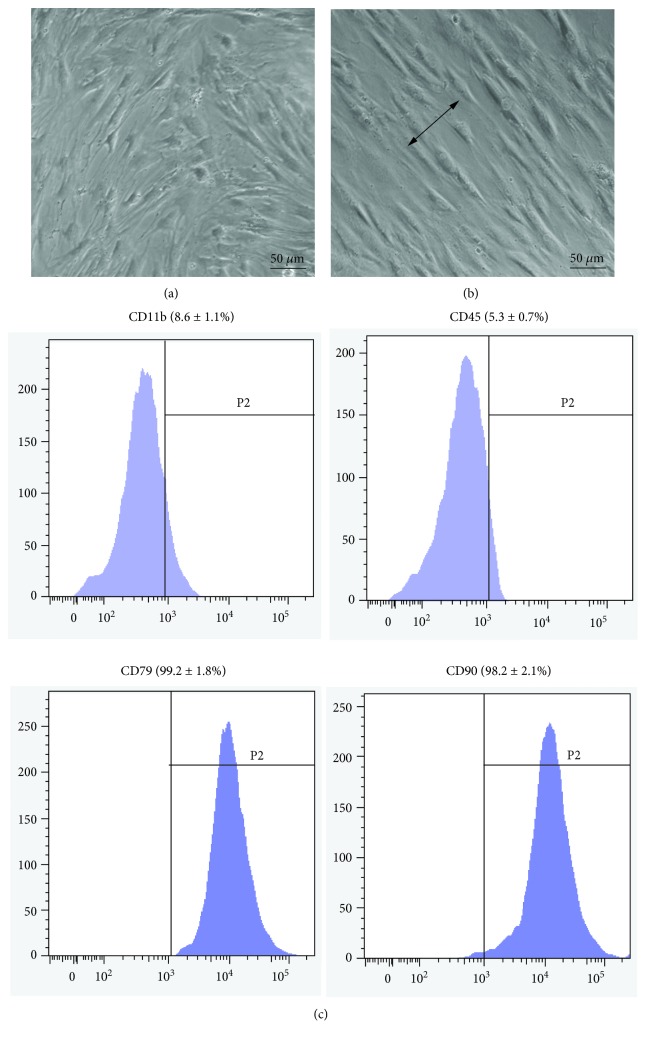
Representative images of BMSCs before and after mechanical stretch. Before mechanical stretch, the obtained cells were fibroblast-like, spindle-shaped, and randomly oriented (a). After mechanical stretch, the BMSCs were still fibroblast-like and spindle-shaped but became much thinner compared to the nonstretch cells. The cells were oriented perpendicularly to the axis of the external strain (b). In addition, immunophenotypic characterization was analyzed by flow cytometry, indicating that the obtained cells were BMSCs (c). The arrow indicates direction of stretch field. Bar = 50 *μ*m.

The obtained cells were fibroblast-like and spindle-shaped (Figure 1(a)). The BMSCs were still fibroblast-like and spindle-shaped after mechanical stretch but were much thinner compared to the nonstretch cells. The cells were oriented perpendicularly to the axis of the external strain, whether in the absence or the presence of applied EPO (Figure 1(b)). In order to evaluate the character of the cells, CD11b, CD45, CD79, and CD90 expressions were analyzed using flow cytometry. The results showed that all cells were positive for cell surface markers CD79 and CD90 with percentages of 99.2 ± 1.8 and 98.2 ± 2.1, respectively; all cells were negative for CD11b and CD45 with percentages of 8.6 ± 1.1 and 5.3 ± 0.7, respectively (Figure 1(c)). These results indicate that the obtained cells were BMSCs.

### 3.2. EPO Promoted the Proliferation of BMSCs

**Figure 2:**
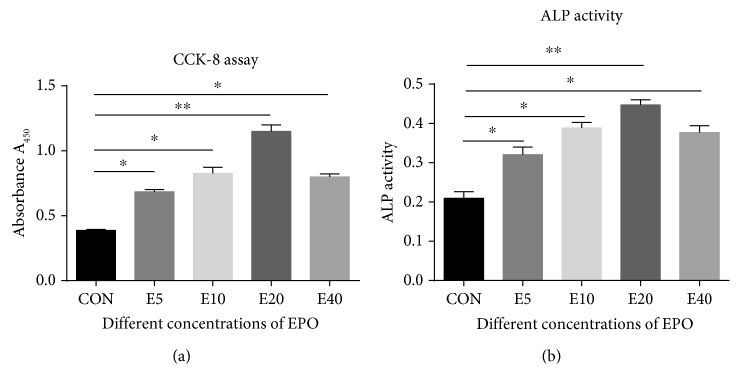
The proliferation and ALP staining of BMSCs under the stimulus of different concentrations of EPO. A dose-response relationship was evident among the experiment groups, and the group of 20 IU/ml EPO showed the maximum value, with the cell proliferation significantly increased about 3-fold compared to the control (a). On the other hand, the staining of the cells treated with EPO is much more obvious than that in the control. Furthermore, the EPO dosage of 20 IU/ml yielded the largest effect relative to the control (b). ^∗^*p* < 0.05 and ^∗∗^*p* < 0.001 compared with the control group.

CCK-8 assay was used to examine the proliferation of the cells. As shown in Figure 2(a), under the stimulus of different concentrations of EPO, a dose-response relationship was evident among the experiment groups, and the group of 20 IU/ml EPO showed the maximum value, with the cell proliferation significantly increased about 3-fold compared to the control (*p* < 0.001). These results indicated that EPO promoted proliferation of BMSCs; meanwhile, the EPO concentration of 20 IU/ml yielded the largest effect.

### 3.3. EPO Promoted the Osteogenic Differentiation of BMSCs

The osteogenic potential of BMSCs was detected using ALP activity. As shown in Figure 2(b), ALP activity of the cells treated with EPO is much higher than in the control. Furthermore, the EPO dosage of 20 IU/ml yielded the largest effect relative to the control. These results indicated that EPO promoted osteogenic differentiation of BMSCs.

### 3.4. Mechanical Stretch Combined with 20 IU/ml EPO Promoted the Proliferation of BMSCs

**Figure 3:**
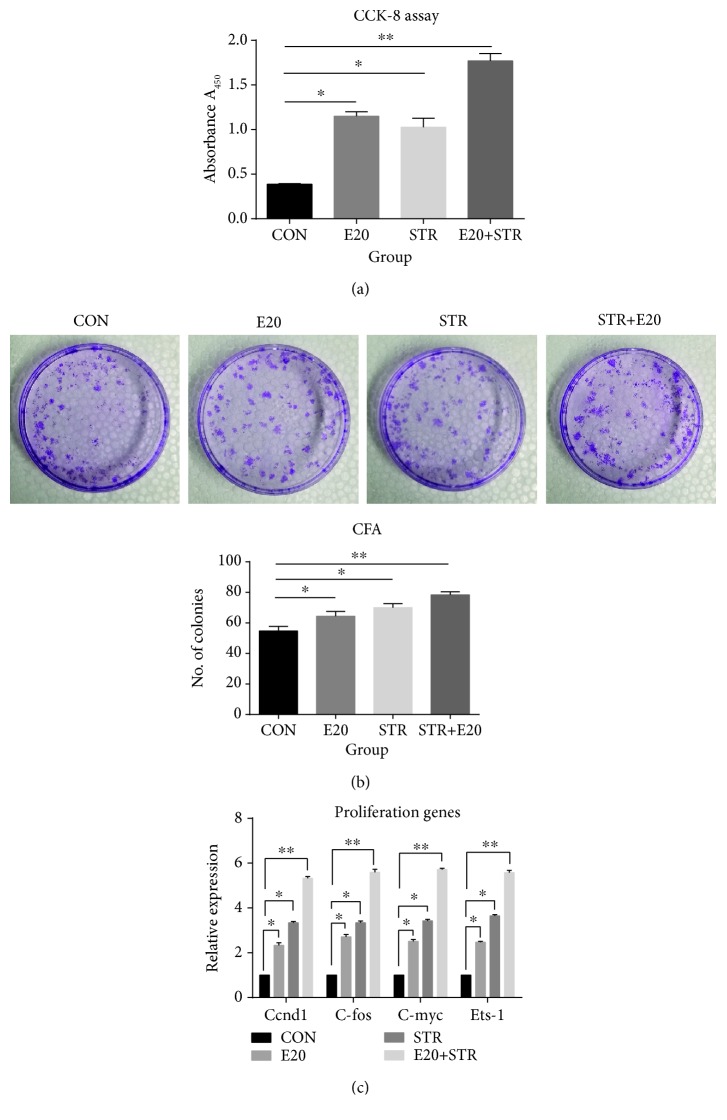
Effects of mechanical stretch combined with 20 IU/ml EPO on the cell proliferation, the distribution of the cell cycles, and the expression of proliferation genes. Under the stimulus of stretch with and without EPO, the cell proliferation increased significantly compared to the control. Among the experiment groups, the STR+EPO group showed the maximum value, with the cell proliferation significantly increased about 4.5-fold compared to the control (a). The number of cell colonies under the intervention of mechanical stretch with and without EPO. One representative well was presented for each group (b). Quantitative results for these assays are also presented as bar graphs. The number of cell colonies that developed was evidently higher under the intervention of mechanical stretch combined with 20 IU/ml EPO compared to the other groups (b). The results showed that mechanical stretch combined with 20 IU/ml EPO stimulated cell proliferation by promoting cell cycle progression from G1 to S-G2/M phases. Besides, qPCR was used to analyze the proliferation genes. BMSCs subjected to mechanical stretch with or without EPO clearly show a change in the mRNA expression levels of Ets-1, C-myc, Ccnd1, and C-fos compared to the control. The mRNA expression of Ets-1 in the group subjected to mechanical stretch combined with 20 IU/ml EPO showed the maximum value, with the expression significantly increased about 5.32-fold compared to the control. The mRNA expressions of C-myc, Ccnd1, and C-fos in the group subjected to mechanical stretch combined with 20 IU/ml EPO increased about 5.59-fold, 5.72-fold, and 5.58-fold, respectively, compared to the control (c). ^∗^*p* < 0.05 and ^∗∗^*p* < 0.001 compared with the control group.

CCK-8 assay was used to examine the proliferation of the cells, both stretched and nonstretched. As shown in Figure 3(a), under the stimulus of stretch whether combined with EPO or not, the cell proliferation increased significantly compared to the control (*p* < 0.01). Among the experiment groups, the STR+EPO group showed the maximum value, with the cell proliferation significantly increased about 4.5-fold compared to the control (*p* < 0.001). These results indicated that mechanical stretch combined with 20 IU/ml EPO promoted proliferation of BMSCs.

To further show the proliferation of BMSCs under the intervention of mechanical stretch whether combined with EPO or not, we investigated the colony-forming characteristic of the cells by evaluating the number of cell colonies that developed after 14 days of being cultured (Figure 3(b)). The number of cell colonies that developed was evidently higher under the intervention of mechanical stretch combined with 20 IU/ml EPO compared to the other groups.

In order to further clarify the role of mechanical stretch combined with 20 IU/ml EPO in regulating the proliferation of BMSCs, qPCR was used to analyze the proliferation genes. As seen in Figure 3(c), BMSCs subjected to mechanical stretch with or without EPO clearly show a change in the mRNA expression levels of Ets-1, C-myc, Ccnd1, and C-fos compared to the control (*p* < 0.01). The mRNA expression of Ets-1 in the group subjected to mechanical stretch combined with 20 IU/ml EPO showed the maximum value, with the expression significantly increased about 5.32-fold compared to the control (*p* < 0.001), as shown in Figure 3(c). The mRNA expressions of C-myc, Ccnd1, and C-fos in the group subjected to mechanical stretch combined with 20 IU/ml EPO increased about 5.59-fold, 5.72-fold, and 5.58-fold, respectively, compared to the control (*p* < 0.001). These results indicated that mechanical stretch combined with 20 IU/ml EPO promoted proliferation of BMSCs through upregulation of the mRNA expressions of Ets-1, C-myc, Ccnd1, and C-fos.

**Figure 4:**
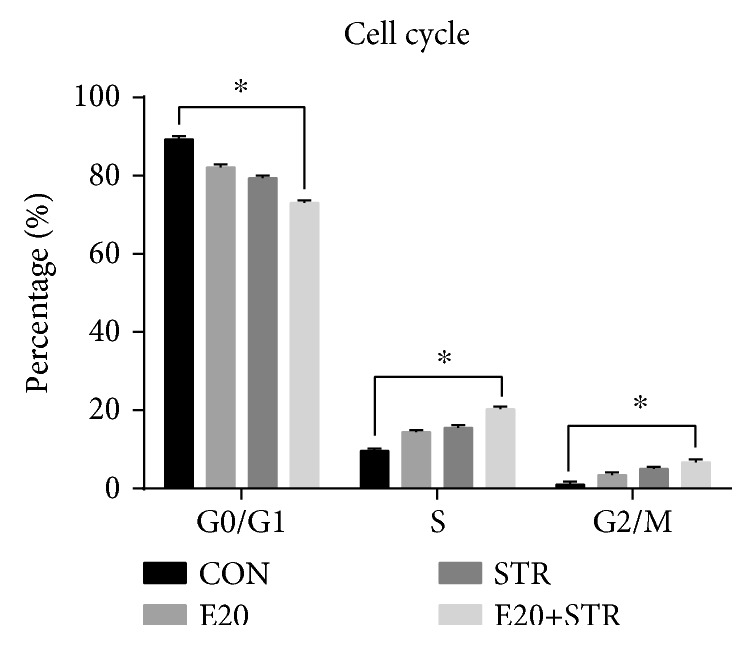
Flow cytometry was used to analyze the distribution of the cell cycles. There was a significantly high S-G2/M phase population with percentages of 27.0 ± 0.74 in the experimental cells (strains combined with 20 IU/ml EPO) compared to the other groups of cells with percentages of 10.7 ± 0.85, 17.9 ± 0.79, and 20.6 ± 0.68 (3B). ^∗^*p* < 0.05 and ^∗∗^*p* < 0.001 compared with the control group.

To understand the process of combining mechanical stretch with 20 IU/ml EPO to regulate the proliferation of BMSCs, flow cytometry was used to analyze the distribution of the cell cycles. As shown in Figure 4, there was a significantly high S-G2/M phase population with percentages of 27.0 ± 0.74 in the experimental cells (strains combined with 20 IU/ml EPO) compared to the other groups of cells with percentages of 10.7 ± 0.85, 17.9 ± 0.79, and 20.6 ± 0.68. The results showed that mechanical stretch combined with 20 IU/ml EPO stimulated cell proliferation by promoting cell cycle progression from G1 to S-G2/M phases.

### 3.5. Mechanical Stretch Combined with 20 IU/ml EPO Promoted the Osteogenic Differentiation of BMSCs

**Figure 5:**
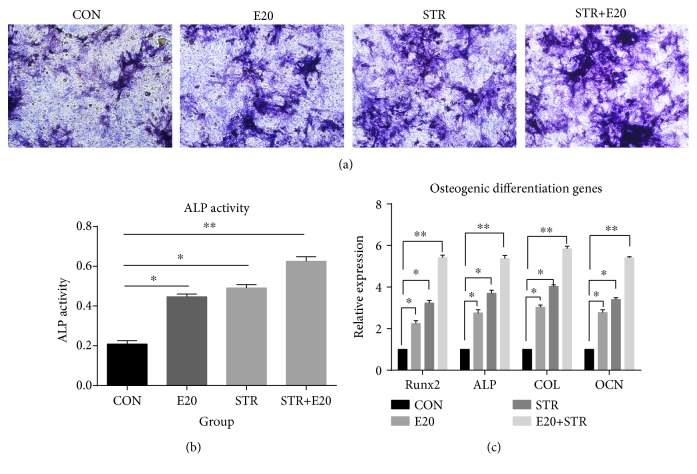
The ALP staining of the cells subjected to mechanical stretch with or without EPO was more obvious than that of the control (a). The group subjected to mechanical stretch combined with 20 IU/ml EPO yielded the largest ALP activity relative to the control (b). BMSCs subjected to mechanical stretch with or without EPO show a change in the mRNA expression levels of ALP, OCN, COL, and Runx2 compared to the control group. The mRNA expression of ALP in the group of mechanical stretch combined with 20 IU/ml EPO showed the maximum value, with the expression significantly increased about 5.37-fold compared to that of the control. The mRNA expressions of OCN, COL, and Runx2 in the group of mechanical stretch combined with 20 IU/ml EPO increased about 5.85-fold, 5.41-fold, and 5.42-fold, respectively, compared to those of the control (c). ^∗^*p* < 0.05 and ^∗∗^*p* < 0.001 compared with the control group.

The osteogenic potential of BMSCs was detected using ALP activity and staining. As shown in Figures 5(a) and 5(b), the activity and staining of the cells subjected to mechanical stretch with or without EPO were more obvious than those in the control. The group subjected to mechanical stretch combined with 20 IU/ml EPO yielded the largest effect relative to the control. These results indicated that mechanical stretch combined with 20 IU/ml EPO promoted differentiation of BMSCs into osteoblasts.

ALP activity and staining is a marker not only of osteoblast differentiation but also of the genes ALP, OCN, COL, and Runx2. In Figure 5(c), we can clearly see that BMSCs subjected to mechanical stretch with or without EPO show a change in the mRNA expression levels of ALP, OCN, COL, and Runx2 compared to the control group (*p* < 0.01). The mRNA expression of ALP in the group of mechanical stretch combined with 20 IU/ml EPO showed the maximum value, with the expression significantly increased about 5.37-fold compared to that of the control (*p* < 0.001), as shown in Figure 5(c). The mRNA expressions of OCN, COL, and Runx2 in the group of mechanical stretch combined with 20 IU/ml EPO increased about 5.85-fold, 5.41-fold, and 5.42-fold, respectively, compared to those in the control (*p* < 0.001). These results indicated that mechanical stretch combined with 20 IU/ml EPO promoted osteogenic differentiation of BMSCs through upregulation of the mRNA expressions of ALP, OCN, COL, and Runx2.

### 3.6. Mechanical Stretch Combined with 20 IU/ml EPO Promoted the Osteogenic Differentiation of BMSCs via the Activation of the ERK1/2 Signaling Pathway

**Figure 6:**
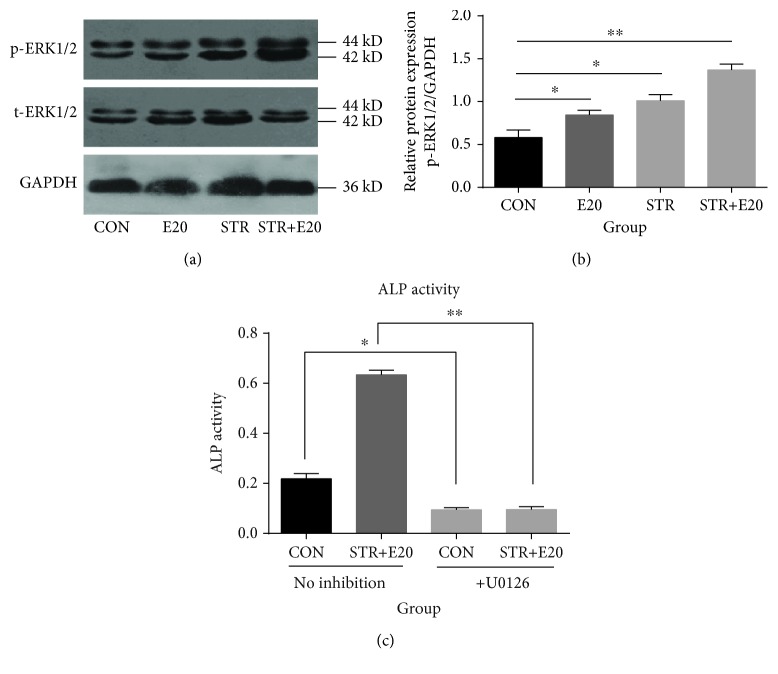
The levels of p-ERK1/2 under mechanical stretch combined with 20 IU/ml EPO. The p-ERK1/2 expression of the cells subjected to mechanical stretch with or without EPO was more obvious than the control. The group subjected to mechanical stretch combined with 20 IU/ml EPO yielded the largest effect relative to the control (a, b). Furthermore, the U0126, an inhibitor of ERK1/2, inhibited the ALP activity induced by mechanical stretch combined with 20 IU/ml EPO (c). ^∗^*p* < 0.05 and ^∗∗^*p* < 0.001 compared with the control group.

The ERK1/2 signal plays a vital role in the osteogenic differentiation of BMSCs; therefore, we analyzed the levels of p-ERK1/2. We found that the p-ERK1/2 expression of the cells subjected to mechanical stretch with or without EPO was more obvious than that of the control. The group subjected to mechanical stretch combined with 20 IU/ml EPO yielded the largest effect relative to the control, as shown in Figures 6(a) and 6(b). Furthermore, the U0126, an inhibitor of ERK1/2, inhibited the ALP activity induced by mechanical stretch combined with 20 IU/ml EPO, as shown in Figure 6(c). These results demonstrated that mechanical stretch combined with 20 IU/ml EPO promoted osteogenic differentiation of BMSCs via activating the ERK1/2 signaling pathway.

## 4. Discussion

It has been determined that EPO promoted the osteogenic differentiation of BMSCs [15–17]. Nevertheless, its effects on the behaviors of BMSCs subjected to mechanical stretch remain unclear. In this study, it was indicated that EPO promoted the proliferation and osteogenic differentiation of BMSCs in a dose-response relationship, with an optimal effect at the dosage of 20 IU/ml. Furthermore, synergistic effect on the proliferation and osteogenic differentiation of BMSCs was obtained from 10% mechanical stretch combined with 20 IU/ml EPO.

In this study, the effects of EPO on the proliferation and osteogenic differentiation of BMSCs were examined with a range of concentrations from 0 IU/ml to 40 IU/ml. Our findings indicated that EPO simultaneously enhanced the proliferation and osteogenic differentiation of BMSCs, with the optimal effects at the dosage of 20 IU/ml. It seems that the effects of EPO depend on not only its concentration but also the type and quantity of BMSCs, and the duration under the treatment as well. Dose-dependent effects of EPO were ever suggested on the osteogenic differentiation of human mesenchymal stromal cells with a range of concentrations from 0 IU/ml to 100 IU/ml, and 20 IU/ml EPO was the lowest effective dose that produced a consistent osteogenic effect on the cells [14]. Kim et al. demonstrated that 20 IU/ml EPO could directly induce the differentiation of human mesenchymal stromal cells into osteoblasts [23]. Nevertheless, in another study, the proliferation effect of EPO was most obvious when the time was 12 hours and the concentration was 5 U/ml [15]. As supposed, with the increase of its concentration, EPO would combine with more erythropoietin receptors (EPORs), leading to a gradually increased effect on the proliferation and osteogenic differentiation of BMSCs. Nevertheless, the high concentration of 40 IU/ml has delayed effect on the proliferation and osteogenic differentiation. It was therefore assumed that there might exist a negative feedback mechanism for the regulation of EPO on the proliferation and osteogenic differentiation when the concentration of EPO amounts to a certain level. This requires further investigation.

The effect of mechanical stretch has been extensively investigated on cell behaviors using various magnitudes. For example, it was reported that mechanical stretch on the order of 1%–3% elongation is needed to obtain a cellular response in vitro [24]. Positive effects were also found using 6% elongation of cyclic mechanical stretch on the osteogenic differentiation of MSCs [25], 8% elongation of strain on the proliferation of rat BMSCs [26], 10% elongation of mechanical stretch on the differentiation of BMSCs [14, 27], and 12% elongation of cyclic stretch on the osteogenesis-related gene expression of osteoblast-like cells [28]. As such, 10% elongation of mechanical stretch was chosen in this study in order to enhance the proliferation and differentiation of BMSCs.

Bone formation following the DO technique is a complex process that involves a series of BMSC activities, including proliferation and osteogenic differentiation [29]. According to the literature, the effect of mechanical stretch on the proliferation of BMSCs is inconsistent. Some studies reported that appropriate mechanical stretch promotes the proliferation of BMSCs [25, 27, 30], whereas many others reported the opposite results [31, 32]. Such discrepancies may be attributable to different strain parameters selected in these studies, including type, magnitude, frequency, and duration. A body of evidence indicates that EPO enhances the proliferation of BMSCs *in vitro* [17, 18] and *in vivo* [19, 20]. In this study, the mRNA expressions of Ets-1, C-myc, Ccnd1, and C-fos of BMSCs, subjected to mechanical stretch with 20 IU/ml EPO, were quantified by qPCR. Ets-1 played a vital role mainly during the first proliferation phase and is also a critical transcription factor in regulating the expression of numerous genes involved in bone development [33]. C-myc is activated by Wnt/*β*-catenin signaling and then induces the proliferation of BMSCs [34]. Moreover, Ccnd1 and C-fos both play important roles in the proliferation of cells, and the activation of these genes could be used as an index of cell proliferation [35, 36]. Our results indicated that the mRNA expression of these genes increased significantly compared to the strain alone, the EPO dosage of 20 IU/ml alone, or the control. In addition, the results of the CCK-8 assay, CFA, and cell cycle assay also supported the aforementioned results. All these results showed that mechanical stretch incorporated with 20 IU/ml EPO significantly promoted the proliferation of BMSCs.

In addition to proliferation, our results indicated that mechanical stretch combined with 20 IU/ml EPO can also enhance the osteogenic differentiation of BMSCs. Osteogenic differentiation of BMSCs is the final and vital step in the process of bone formation. As shown, the Runx2 protein has the ability to bind to and regulate the expression of other genes, including those for collagen type I, osteocalcin, and osteopontin [37], and it is necessary for membranous bone formation [38]. Several studies have reported that Runx2 is a mechanical stretch target gene that increases osteoblastic activity [39, 40]. Meanwhile, Shiozawa et al. proved that when BMSCs were treated with EPO, the mRNA expression of Runx2, the mineral deposition, and the ALP activity were enhanced [41]. In addition to Runx2, ALP, OCN, and COL I are also typically used in the identification of osteogenic differentiation of cells [30, 42, 43]. Our result is consistent with those mentioned above; additionally, among the experiment groups, the group of mechanical stretch combined with 20 IU/ml EPO showed the maximum value.

However, the real mechanism of mechanical stretch combined with EPO affecting the osteogenic differentiation of BMSCs remains to be discovered. EPO, a hypoxia-regulated factor, combined with different kinds of cells through EPORs and elicited a variety of reactions in the cells [44–46]. There are EPORs in the surface of BMSCs, and the EPO can activate the ERK1/2 signaling pathway. Previous studies also confirmed that mechanical stretch can activate the ERK1/2 signaling pathway [39, 44], and the ERK1/2 signal plays a vital role in the osteogenic differentiation of BMSCs. One hypothesis is that when BMSCs are subjected to mechanical stretch, the stretch may induce the cells into a hypoxic state [41, 44]; as a result, cells may have a stronger ability to combine with EPO. Meanwhile, the application of EPO would activate the ERK1/2 signaling pathway, the same function of mechanical stretch on the cells; therefore, stretch and EPO may work synergistically, activating the ERK1/2 signaling pathway and finally promoting the osteogenic differentiation of BMSCs. Additionally, consistent with previous studies [30, 47], BMSCs in this study were observed to be oriented perpendicularly to the stretch axis after applied stretch (10% elongation). Cell function and cell fate are often determined by cell shape. Cells in different native tissues often show different specific morphologies, such as spindle tendon cells, dendritic neuronal cells, or flattened osteogenic cells. Cell fate is primarily determined by its morphology. For example, at the same occupying area, BMSCs with a sharp pentagon and high aspect ratio exhibit higher cell contractility, thus leading preferentially to osteogenic differentiation [48, 49]. Therefore, with an appropriate parameter of mechanical stretch, the F-actin filaments in BMSCs would become more arranged and oriented perpendicular to the axis of mechanical loading [47], then change cells' morphology, and finally lead preferentially to osteogenic differentiation.

Mechanical stretch on cells in vitro is somehow like the DO technique in vivo. This work demonstrated that an appropriate parameter of mechanical stretch (10%, 1 Hz, twice every day, for 4 h for each treatment, 7 days) enhances the proliferation, migration (data not shown), and differentiation of BMSCs. This combination may provide an effective therapeutic strategy for posttraumatic complex nonunion with shortening treatment duration, decreasing complications and reducing hospital costs. Nevertheless, we should acknowledge that there are some limitations to this study. First, the parameter of mechanical stretch used in this study needs to be optimized, although the positive effect was noted. Further investigations are needed to understand the best parameters for the osteogenesis of BMSCs. Second, this study found that 20 IU/ml EPO yielded the largest effect on BMSCs, but the concentration of EPO between 20 IU/ml and 40 IU/ml might yield a larger effect if combined with higher numbers of EPORs. Third, this study found that mechanical stretch promoted the osteogenic differentiation of BMSCs induced by erythropoietin via activating the ERK1/2 signaling pathway, but the upstream and downstream factors involved in the ERK1/2 signaling pathway remain unclear. Finally, the *in vivo* experiment is necessary to further determine the effects of mechanical stretch combined with EPO on bone formation.

## 5. Conclusion

In summary, our findings suggested that EPO was able to promote the proliferation and osteogenic differentiation of BMSCs, and these effects were enhanced when combined with mechanical stretch. The underlying mechanism may be related to the activation of the ERK1/2 signaling pathway.

## Figures and Tables

**Table 1:** Primer sequences for qPCR.

Gene	Primer	Primer sequence (5′-3′)	Product (bp)
C-fos	Forward	TGCATGAATTCCCCAGCCGACTC	618
	Reverse	TGCATAAGCTTCAGCTCCCTCCT	
Ets-1	Forward	GAGTTCAGCCTGAAGGGTGTT	153
	Reverse	CACATCCTCTTTCTGCAGGATCT	
C-myc	Forward	AATTCCAGCGAGAGACAGAG	434
	Reverse	CAAAGCCCTTCTCACTCCA	
Ccnd1	Forward	CGTACCCTGACACCAATCTC	434
	Reverse	TGAAGTAAGAAACGGAGGGC	
ALP	Forward	TATGTCTGGAACCGCACTGAAC	90
	Reverse	CACTAGCAAGAAGAAGCCTTTGG	
OCN	Forward	GCCCTGACTGCATTCTGCCTCT	192
	Reverse	TCACCACCTTACTGCCCTCCTG	
COL	Forward	CAGGCTGGTGTGATGGGATT	278
	Reverse	CCAAGGTCTCCAGGAACACC	
Runx2	Forward	ATCCAGCCACCTTCACTTACACC	199
	Reverse	GGGACCATTGGGAACTGATAGG	
GAPDH	Forward	TGCCACTCAGAAGACTGTGG	129
	Reverse	TTCAGCTCTGGGATGCCTT	

## Data Availability

The data used to support the findings of this study are included within the article.
